# Retraction of rod-like mitochondria during microtubule-dependent transport

**DOI:** 10.1042/BSR20180208

**Published:** 2018-06-29

**Authors:** María Cecilia De Rossi, Valeria Levi, Luciana Bruno

**Affiliations:** 1 Departamento de Química Biológica, Facultad de Ciencias Exactas y Naturales, Universidad de Buenos Aires, IQUIBICEN-CONICET, Ciudad Universitaria, Ciudad de Buenos Aires CP1428, Argentina; 2 Departamento de Física, Facultad de Ciencias Exactas y Naturales, Universidad de Buenos Aires, IFIBA-CONICET, Ciudad Universitaria, Ciudad de Buenos Aires CP1428, Argentina

**Keywords:** intracellular transport, mitochondria, molecular motors, Xenopus laevis melanophores

## Abstract

Molecular motors play relevant roles on the regulation of mitochondria size and shape, essential properties for the cell homeostasis. In this work, we tracked single rod-shaped mitochondria with nanometer precision to explore the performance of microtubule motor teams during processive anterograde and retrograde transport. We analyzed simultaneously the organelle size and verified that mitochondria retracted during retrograde transport with their leading tip moving slower in comparison with the rear tip. In contrast, mitochondria preserved their size during anterograde runs indicating a different performance of plus-end directed teams. These results were interpreted considering the different performance of dynein and kinesin teams and provide valuable information on the collective action of motors during mitochondria transport.

## Introduction

Intracellular transport relies on molecular motors that step along cytoskeletal filaments dragging cargoes through the crowded cytoplasm (reviewed in [1,2]).

Different lines of research in a variety of cell types have demonstrated that microtubule-dependent motors team up during intracellular transport. The team performance depends on the biophysical properties of the motors (e.g. [3–5]), the number of motors participating in the team [6,7], and their mechanical coupling when attached to the cargo surface [8–10] amongst other factors.

Cargoes moving along microtubules present frequent switches on their direction due to the presence of kinesin and dynein motors on its surface; these motors pull the cargo toward the microtubule plus- and minus-end, respectively and the strongest team determines the cargo direction (e.g. [3,11–13]). The selective motor recruitment to specific cargoes [14] and their interactions with regulatory proteins (reviewed in [15]) also bias the natural tug of war and contribute to define the organelle directionality (reviewed in [16]). Despite years of research in this field have improved our knowledge on the function of molecular motors, we still do not know many relevant aspects of the collective action of motors during intracellular transport.

Particularly, previous works on collective transport mainly focussed their attention on multiple motor copies attached to spherical-shaped cargo (e.g. [6,17]). The geometrical constraints defined by the cargo’s shape may impact the distribution of motors on the cargo and also influence how the drag is shared between microtubule-attached motors. Therefore, it is extremely relevant to explore how motors work together when transporting cargoes with a different geometry in living cells.

Amongst other cargoes, motors contribute to regulate the intracellular organization of mitochondria, organelles responsible for the generation of the ATP required in numerous cellular processes. These organelles also have fundamental roles in lipid metabolism, calcium homeostasis, cell differentiation amongst many other processes (reviewed in [18,19]); a correct positioning of these organelles is essential for relevant processes such as cell division and migration [20].

Mitochondria are fascinating organelles from a biophysical perspective since they form an interconnected network with cell-type specific morphologies that span from small rounded vesicles to branched tubular networks (reviewed in [20,21]). Even in a single cell, the mitochondrial network is continuously remodeled as a consequence of events of fission, fusion, and motility. The rates of these processes respond to certain cellular cues such as changes in the oxidative metabolism, calcium homeostasis, and apoptotic or necrotic cell death [20–22]. For example, cellular stresses such as DNA damage increases the fusion rate generating larger and connected mitochondria; it is proposed that this process allows mixing up the components of functional and dysfunctional mitochondria rescuing the damaged mitochondria [20,21].

Molecular motors seem to play a relevant role in the regulation of mitochondria size and shape [23–26], properties that have been proved to be essential for cell homeostasis [21]. It has been suggested that changes in mitochondria morphologies that end up in rod-like shapes would facilitate the transportation of these organelles along microtubules tracks [27].

Microtubule-dependent motors anchor to mitochondria through adaptor proteins such as the Miro–Milton complex and KBP that modulate the number of motors attached to the cargo and/or their activity (reviewed in [28]) thus affecting the transport performance. It has been proposed that mitochondria may attach the plus-end directed motors kinesin-1 and kinesin-3 and the minus-end cytoplasmic dynein [25,29,30].

In this work, we explored the performance of microtubule-motor teams focussing our analysis on the transport of rod-shaped mitochondria in *Xenopus laevis* melanophores. These cells are relatively big (~50 μm) and thin (~3 μm, [31]); therefore, the pathway of these organelles along microtubules in the cell periphery are essentially bidimensional.

Rod-shaped mitochondria are attractive to explore the performance of motor-teams since their elongated shape determines a broad distribution of motors on the surface. As we mentioned before, the mechanical communication amongst active motors is expected to be different from that observed in small, rounded organelles as peroxisomes [8] where active motors are spatially confined to a smaller area and thus they are probably closer to each other.

In this work, we tracked mitochondria with subpixel resolution and analyzed whether the organelles change their size during processive transport along microtubules. We verified a correlation between mitochondria retraction and retrograde mitochondria transport; this result could be explained considering the different performance of anterograde and retrograde motor’s teams. Our results contribute to a better understanding of the mechanisms involved in the organization of mitochondria in cells.

## Materials and methods

### Cell culture and sample preparation for imaging

Immortalized *Xenopus laevis* melanophores stably expressing the *Xenopus* homolog of τ protein XTP fused to EGFP [32,33] were cultured as described in [34]. Cells were grown in 70% L-15 medium (Sigma-Aldrich) supplemented with phenylthiourea [35] to reduce the amount of melanosomes. For microscopy measurements, cells were plated for 2 days on 25-mm circular coverslips placed into 35-mm dishes in 2.0 ml culture medium.

Before observation, the coverslips were washed with serum-free medium, mounted in a custom-made chamber specially designed for the microscope and incubated with 10 mM Latrunculin B (Sigma-Aldrich) for 30 min to depolymerize actin filaments. Mitochondria were labeled adding 100 nM MitoTracker Deep Red FM (Invitrogen) to the incubation medium.

### Confocal imaging

Confocal images were acquired in an FV1000 confocal microscope (Olympus Inc.). EGFP-XTP and MitoTracker Deep Red FM were observed using a multi-line Ar laser tuned at 488 nm and a 635-nm diode laser as excitation sources (average power at the sample, 2 and 0.2 μW, respectively). The laser’s light was reflected by a dichroic mirror (DM 405/488/543/635) and focussed through an Olympus UPlanSApo 60× oil immersion objective (NA = 1.35) on to the sample. Fluorescence was collected by the same objective and split into two channels set to collect photons in the range 500–525 nm (EGFP) and 650–750 nm (MitoTracker Deep Red FM). Fluorescence was detected with photomultipliers set in the photon-counting detection mode.

Time-lapse confocal movies (100–150 frames, pixel size = 63 nm) of individual fluorescent mitochondria were collected at a speed of 0.6 frames/s.

### Mitochondria tracking

Custom-made routines developed with Matlab’s Image Processing Toolbox (The Mathworks Inc.) were used to obtain the subpixel centroid position and morphological properties of mitochondria in a semi-automatized way.

Briefly, the user starts the analysis by choosing a mitochondrion by clicking on top of its image in the first frame of the movie. Then, each frame is binarized using the adaptive intensity threshold command *background*. The centroid of the organelle and its main axis length were recovered in each frame applying the *regionprops* function. To check the performance of the routine, we simulated time-lapse images of mitochondria with varying S/N as described previously [36] and found that the errors on the centroid position and on the organelle length were 3–20 nm and 10–40 nm, respectively for S/N in the range 30–100, i.e. in the order of those found in the experiments.

We considered that the mean direction of transport is given by the shape of the centroid trajectory and calculated this direction by fitting a second order polynomial to each mitochondria trajectory, as reported in [37]. The centroid trajectory was further decomposed into parallel and perpendicular motion with respect to the transport direction axis; the former is essentially the distance travelled along the filament as a function of time. These data were further classified into pauses and periods of directed motion or runs where the organelle moves unidirectional for at least 250 nm. The mean run speed was determined by linear regression of the distance compared with time data.

The mitochondrial length variation during a run (ΔL) was determined as the difference between L values registered at the beginning (L_o_) and the end (L_f_) of the run. The organelle was considered to change the size when |ΔL| > 100 nm.

The positions of the organelle’s extremes (i.e. tips) were assigned from the borders of the binary image of the organelles determined using the *regionprops* function of Matlab. The mean speeds of the leading and rear tips were then obtained calculating the distance travelled by the tips during the run period. The statistical analysis of these data was performed using the one-sample *t-*test.

### Statistical analyses

A Chi-square test for homogeneity [38] was used to test differences on the frequencies of certain *i* events, particularly the preservation of the size, retraction, or elongation of mitochondria during anterograde and retrograde transport.

Briefly, the test consists of determining the value of the statistic:

(1) $${\text{χ}}^{2}=\sum_{\text{i}}\frac{{\left({\text{O}}_{\text{i}}-{\text{E}}_{\text{i}}\right)}^{2}}{{\text{E}}_{\text{i}}}$$

where O_i_ and E_i_ are the observed and expected frequency of the *i* event during anterograde and retrograde transport. The *P*-value for this test is computed as:

(2) $$p-value=p({\text{χ}}_{2}^{2}>{\text{χ}}^{2})$$

where ${\text{χ}}_{2}^{2}$ is the Chi-square distribution with two degrees of freedom.

Frequencies were considered significantly different from each other when the null hypothesis, i.e. both distributions are the same, can be rejected (*P*-value <0.005).

## Results

Melanocytes expressing XTP-EGFP, a tau-like protein from *Xenopus laevis* were treated with latrunculin to depolymerize actin filaments and labeled afterward with MitoTracker Deep Red FM. Confocal, two-color images of the cells (Figure 1A) show XTP-EGFP labeled microtubules in close contact with mitochondria with various morphologies including diffraction-limited dots, rods, branched rods, and loops as observed in other cell systems [39]. Time-lapse images showed that some of the organelles fluctuate around fixed positions, whereas others present periods of fast and processive motion along microtubules (Figure 1B–D). Supplementary Movie S1 also shows that only rounded and rod-like mitochondria exhibited processive motion along MTs in agreement with previous works suggesting that mitochondria require elongated morphologies to be transported along the cytoskeleton [27].

**Figure 1:**
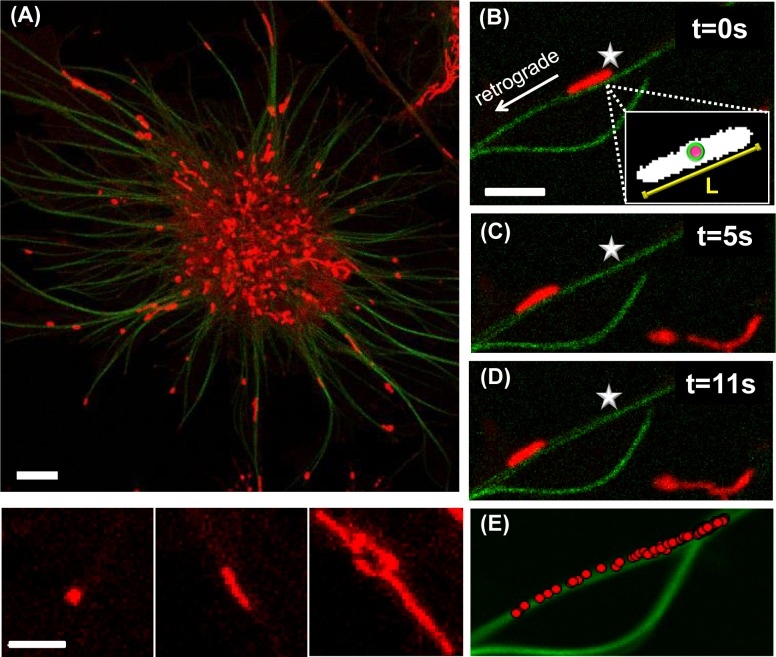
Tracking mitochondria in cells (**A**) Representative confocal image of *X. laevis* melanocyte expressing EGFP-XTP (green) incubated with MitoTracker Deep Red (red) reveals the variability in mitochondrial morphology (bottom panel). Scale bars: 10 and 3 μm, respectively. (**B**–**E**) Representative images obtained during a time-lapse movie of rod-like mitochondria. The frames were analyzed as described in the text to determine the organelle centroid position and main axis length. Inset: binary image of the organelle showing the centroid position (colored circle) and organelle length (yellow line). Scale bar: 3 μm. (E) The mitochondria centroid position (red circles) followed the mean position of the microtubule recovered in the movie (green).

To recover the trajectories of these organelles with high spatial precision, we tracked the centroid of rod-like mitochondria moving along microtubules as described in ‘Materials and methods’ section (Figure 1B–E) and fitted the trajectory with a second order polynomial function to define the main transport axis. This axis practically overlapped with the microtubule co-ordinates (Figure 1E). The trajectory was further decomposed to determine the distance travelled along the microtubule as a function of time. In order to test whether the organelle changed its size during periods of active motion, we also determined the length of the mitochondria at every frame of the movie and calculated the length variation ΔL as described in ‘Materials and methods’ section.

Figure 2A shows representative data obtained from a tracking experiment; in this particular case the mitochondrion contracted ~30% during retrograde transport and extended during transport in the opposite direction recovering its initial size. These observations are in-line with previous data showing that rod-shaped mitochondria may change their lengths during transport [39].

**Figure 2:**
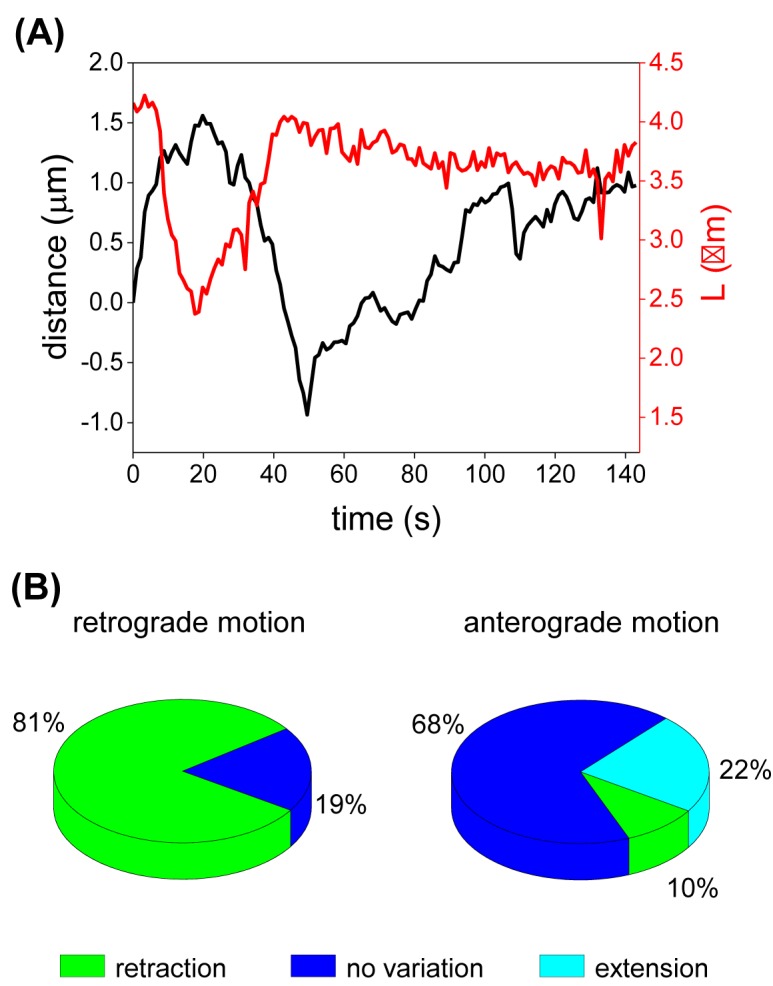
Mitochondial length variation during transport (**A**) Representative example of a mitochondrion main axis length (L, red) and its trajectory along the microtubule (black). (**B**) Quantitation of mitochondria length variation during directed motion. L values registered at the beginning (L_o_) and at the end (L_f_) of retrograde (*n*=52) and anterograde (*n*=62) runs were determined whether the mitochondria experienced a retraction, extension, or no variation in their length. The figure includes data of 75 mitochondria (*n*_cells_=35).

To test whether the retraction and extension processes correlate with the organelle direction, we split the trajectories into pauses and periods of unidirectional motion or runs (see ‘Materials and methods’ section) and classified these runs according to the organelle direction. Supplementary Figure S1 showed that anterograde and retrograde run lengths were similar; in contrast, rod-like retrograde organelles moved faster than anterograde mitochondria. This different behavior was not related to the size of the organelles since the long axis of anterograde and retrograde organelles were similar (Supplementary Figure S1). The higher speed of retrograde organelles probably reflects a better performance of the minus-end directed teams during transport of rod-like mitochondria in the crowded intracellular milieu as discussed below.

Figure 2B shows that most anterograde mitochondria preserve their size during transport while comparatively smaller numbers of organelles either retract or extend. In contrast, ~80% of organelles retracted when moving toward the microtubule minus-ends. These results suggest that anterograde and retrograde teams present a different behavior when transporting elongated mitochondria in the crowded cytoplasm (*P*-value < 0.005).

We next focussed on the properties of the retraction process during retrograde transport and observed that the end of the retraction always occured simultaneously with either reversions or pauses in the trajectories (100% of the events, *n*_retraction events_=33) suggesting that the end of the retraction is due to the same processes that terminate retrograde runs, e.g. the stochastic disengagement of the retrograde team also triggered by opposed-polarity motors or encounters with obstacles. In addition, the relative retraction of mitochondria during retrograde transport followed an exponential-decay distribution (Figure 3A) and indicates that mitochondria reduces ~13% its size during retrograde transport.

**Figure 3:**
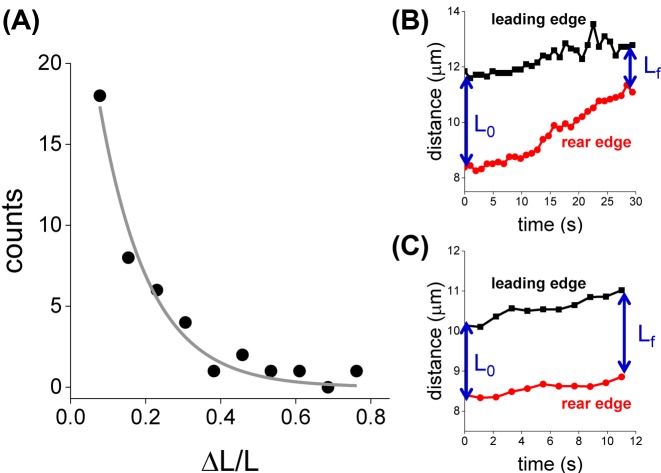
Properties of mitochondria retraction during retrograde transport (**A**) Distribution of the relative mitochondria length variation during retrograde runs (*n*_runs_=42). The data were fitted with an exponential-decay function (line) obtaining a characteristic retraction length of 13 ± 1%. The figure includes data of 31 mitochondria (*n*_cells_=21). (**B**,**C**) Representative trajectories of the mitochondria leading and rear edges obtained during retrograde (B) and anterograde (C) runs.

Several experimental and theoretical works showed that motor carrying relatively big cargoes do not share the load equally (e.g. [6,40,41]); particularly, leading motors assume more load than trailing motors. Therefore, we decided to follow the motion of the leading and rear tips of retrograde mitochondria and quantitated the ratio between their speeds to recover information regarding the performance of leading and trailing teams during the retraction process. Figure 3B shows that the mitochondria rear-end moved significantly faster (*P*-value=0.01), with a speed ~30% higher than the corresponding leading-end suggesting that the contribution of individual motors to the team performance depends on their relative position on the organelle.

We also performed a similar analysis for anterograde mitochondria (Figure 3C) and observed that the organelle extremes showed similar velocities suggesting a similar behavior of the leading and trailing teams during anterograde transport of mitochondria.

## Discussion

The distribution of mitochondria, their shape, and connectivity are fundamental for the correct development of many cellular functions [28] and requires their active transport along cytoskeletal tracks. In addition, motors influence the mitochondrial fission and fusion processes [24] that are required to maintain a population of healthy mitochondria in neurones. Impairment of these processes in diseases such as Alzheimer’s, Parkinson’s, and Huntington’s [42] stresses the necessity of understanding mitochondria dynamics.

In this work, we have studied the transport of rod-like mitochondria along microtubules and found a strong correlation between retrograde transport and mitochondria retraction. We also found that most anterograde mitochondria preserved their size during transport clearly showing that the mechanical communication amongst motors in minus-end and plus-end teams is different.

To explain these differences, we consider relevant and well-known biophysical properties of dynein and kinesin motors. Specifically, we take into account that: (i) the dense cytoplasmic environment restrains the motion of micrometer-sized cargoes introducing an important drag on motors [43,44]; (ii) motors dragging the organelle do not share the load equally with those located at the leading edge subject to a higher load than rear motors [6,40,41] and; (iii) teams of dyneins or kinesins respond differently to the drag probably due to their different single-motor properties. The latter statement was proposed by Rai et al. [17] who showed that the velocity of kinesin decreases with the load in a convex-up manner whereas dynein presents a concave-up behavior. The authors suggest that the initial stepper reduction in the force-velocity (F-V) curve of dynein in comparison with kinesin’s could be a consequence of the different stepping behavior of the motors since the step size of dynein decreases with the load whereas kinesin step size does not depend on the load [4].

Figure 4A outlines a model built to explain the retraction observed during mitochondria retrograde transport. We propose that dyneins located at the mitochondrial leading-end work against a higher load than those attached to the rear end; as a consequence of the concave-up F-V curve of dynein described previously, the rear team moves faster as verified in Figure 3B and causes the retraction of the organelle. This model is in line with Rai et al. [17] that proposed that leading dyneins in a load-carrying team take short steps, whereas trailing dyneins take larger steps. In this context, the rear team moves faster and causes the mitochondrion retraction.

**Figure 4:**
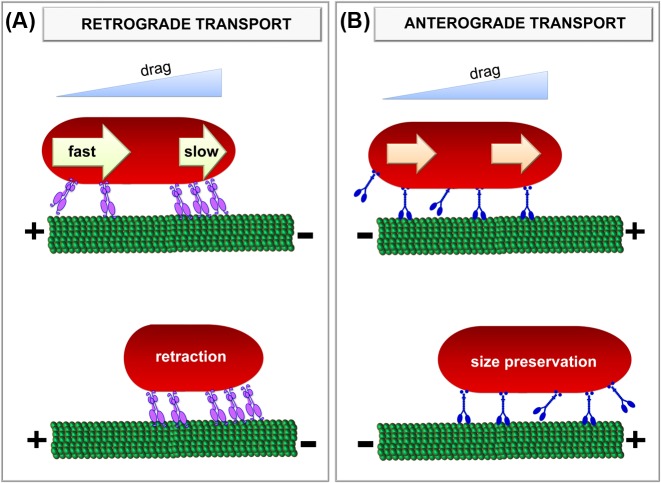
Graphical summary depicting the influence of microtubule motors teams on rod-like mitochondria size during processive transport The opposed-polarity motors kinesin (blue) and dynein (violet) stochastically attach and detach from the microtubule and pull the cargo against the drag force imposed by the cytoplasm. The schemes shows arbitrary configurations and number of motors attached to mitochondria. To simplify the scheme, we did not include opposed-polarity motors that are also attached to the organelle and/or competing with the motors responsible for the transport during unidirectional runs. (**A**) During retrograde transport, the leading dyneins work against a higher load and thus move slower than those attached at the rear-end; as a consequence, the mitochondrion retracts. (**B**) During anterograde transport, kinesins asynchronous step along the track switching from an active state to inactive (and *vice versa*). Due to the convex-up F-V curve of kinesins, leading and trailing motors present similar speeds and thus the mitochondrion does not change its size.

Figure 4B shows the behavior expected for kinesin teams during processive anterograde transport of mitochondria. Given the inability of kinesins to cooperate with each other (e.g. [17]), we proposed that only a few kinesin motors pull the cargo at any moment. Despite the drag force at the leading tip of the mitochondria is expected to be higher, leading and trailing motors move at similar velocities as a consequence of the convex-up F-V curve of this motor. This model could explain the conservation of the organelle size observed for most mitochondria during anterograde transport (Figure 2B).

Taking together, our results show the different behavior of plus and minus-end directed teams during intracellular transport of mitochondria. We believe that the present study advances on our knowledge of the mechanisms of collective motor function and may be important to understand how motor teams work in processes such as fission and fusion which are essential for mitochondrial homeostasis.

## Supporting information

**Figure S1.** **(A)** Representative distribution of L values obtained for retrograde and anterograde rod-like mitochondrias. **(B)** The organelles trajectories (N=75) were analized as described in the text to recover segments of directed motion (runs). The run length median values and the mean speed experienced by the mitochondria along these runs were computed. The data is expressed with the standard error.

**Supplementary Movie S1.** Time-lapse confocal movie of a *X. laevis* melanocyte expressing EGFP-XTP (green) incubated with MitoTracker Deep Red (red). The movie was acquired at a speed of 0.1 frame/s during 295 s. The arrows point some mitochondria processively moving along microtubules.
