# Supplementary material for: Retraction of rod-like mitochondria during microtubule-dependent transport
Source: Biosci Rep. 2018 Jun 29;38(3):BSR20180208. doi: 10.1042/BSR20180208 (PMC6013701; doi:10.1042/BSR20180208)
Supplement: Supplementary file 1 [file bsr20180208_Supp1.pdf]

Supplementary Material

A

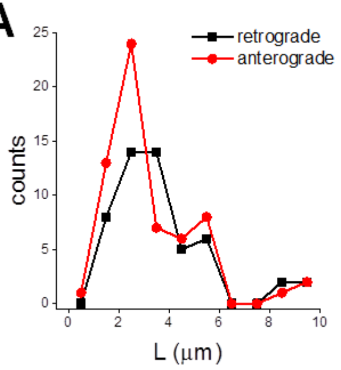

B

parameter	retrograde motion	anterograde motion
run length (nm)	1570 ± 370	1350 ± 270
mean speed (nm/s)	173 ± 30	108 ± 10

Figure S1. (A) Representative distribution of L values obtained for retrograde and anterograde rod-like mitochondrias. **(B)** The organelles trajectories (N=75) were analized as described in the text to recover segments of directed motion (runs). The run length median values and the mean speed experienced by the mitochondria along these runs were computed. The data is expressed with the standard error.

Supplementary Movie S1. Time-lapse confocal movie of a *X. laevis* melanocyte expressing EGFP-XTP (green) incubated with MitoTracker Deep Red (red). The movie was acquired at a speed of 0.1 frame/s during 295 s. The arrows point some mitochondria processively moving along microtubules.
